# Growth and Characterization of Cu_2_Zn_1−x_Fe_x_SnS_4_ Thin Films for Photovoltaic Applications

**DOI:** 10.3390/ma13061471

**Published:** 2020-03-24

**Authors:** Vanira Trifiletti, Giorgio Tseberlidis, Marco Colombo, Alberto Spinardi, Sally Luong, Mati Danilson, Maarja Grossberg, Oliver Fenwick, Simona Binetti

**Affiliations:** 1 School of Engineering and Materials Science, Queen Mary University of London, 327 Mile End Road, London E1 4NS, UK; v.trifiletti@qmul.ac.uk (V.T.); s.luong@qmul.ac.uk (S.L.); o.fenwick@qmul.ac.uk (O.F.); 2 Department of Materials Science and Solar Energy Research Center (MIB-SOLAR), University of Milano-Bicocca, Via Cozzi 55, I-20125 Milano, Italy; giorgio.tseberlidis@unimib.it (G.T.); m.colombo224@campus.unimib.it (M.C.); a.spinardi@campus.unimib.it (A.S.); 3 Department of Materials and Environmental Technology, Tallinn University of Technology, Ehitajate Tee 5, 19086 Tallinn, Estonia; mati.danilson@taltech.ee (M.D.); maarja.grossberg@taltech.ee (M.G.)

**Keywords:** sustainable energy, chalcogenide solar cells, kesterite, stannite, iron chalcogenide

## Abstract

Photovoltaics is a promising technology to produce sustainable energy, thanks to the high amount of energy emitted by the sun. One way of having solar cells with low production costs is to apply thin-film technology and with earth-abundant raw materials. A keen interest is arising in kesterite compounds, which are chalcogenides composed of abundant and non-toxic elements. They have already achieved excellent performance at the laboratory level. Here, we report the synthesis and characterization of mixed chalcogenides based on copper, zinc, iron, and tin. Solutions have been studied with different zinc and iron ratios. The distortion of the elementary cell of kesterite increases with the addition of iron until a phase transition to stannite occurs. The process of synthesis and deposition proposed herein is cheap and straightforward, based on the sol-gel technique. These thin films are particularly attractive for use in cheap and easily processable solar cells. The synthesized layers have been characterized by X-ray diffraction, UV-Vis absorption, and Raman, X-ray photoelectron, and energy-dispersive X-ray spectroscopy measurements.

## 1. Introduction

For decades, the most developed economies have been pushing toward the abandonment of fossil fuel technologies in favor of technologies that use renewable energy [1]. The production of electricity from renewable sources, of which photovoltaics contributes with less than 10%, exceeded 26% of total production in 2018 [2]. The research on solar cells is driven by the enormous amount of solar radiation that reaches the Earth, which is 100 million times the total energy consumed in one year [3]. Moreover, solar panels can be installed on any roof and so, being a decentralized technology, can supply energy directly to consumers [4]. One way for producing high-efficiency solar cells with low production costs is to employ thin-film technology, which uses nanometer- or micrometer-thick layers to build a device. In this scenario, quaternary chalcogenides have aroused great attention, above all, for the thickness of the solar cells, which is usually around 2μm. The CuIn_1−x_GaxSe_2_ (CIGS) thin film stands out in this family, being able to compete in efficiency with the well-established silicon technology [5]. However, CIGS has the disadvantage of being composed of indium and gallium, which are rare elements and not suitable for large-scale production [6]. An alternative is to replace indium and gallium with zinc and tin, selenium with sulfur, moving to the kesterite Cu_2_ZnSnS_4_ (CZTS), or by replacing zinc with iron and moving to the stannite phase structure Cu_2_FeSnS_4_ (CFTS). In nature, this compound is found as a mineral in which zinc and iron are mixed, and it takes on two compositions: Kesterite with less than 30% of iron, and stannite with an iron percentage exceeding 80% [7]. Altering the phase structure also changes the optical properties, so the iron-to-zinc ratio controls the energy gap that can be tuned between 1.36 and 1.51 eV [8]. Like the other chalcogenides, these compounds also have a high absorption coefficient and a direct bandgap, so the final thickness of the absorber material can be minimal, in the micrometer range, with a theoretical photovoltaic energy conversion limit greater than that of silicon (30–32%) [9]. Nevertheless, CZTS-based solar cells have only recently exceeded 10% laboratory efficiency [10], and CFTS-based ones do not even reach 5% efficiency [8]. Most of the research on CFTS is focused on the synthesis of nanoparticles, to be later deposited as thin films; however, such layers suffer, above all, from bad adhesion to the substrate and uneven grain growth [8]. To overcome these drawbacks, the most common deposition techniques for solution-processable materials, such as blade coating, spin coating, drop casting, or spray coating, should be employed to prepare uniform and compact CFTS thin films. Nevertheless, despite being a very promising compound [6], very little progress has been made in optimizing CFTS thin films; therefore, a more detailed understanding of iron-based chalcogenides as a solar radiation absorbent layer is required [8].

Cu_2_Zn_1−x_Fe_x_SnS_4_, C(Z,F)TS, literature until 2014 reports that the transition from kesterite to stannite occurs with about 40% iron [11,12]. In 2016, Shadrokh et al. [13] argued that the phase transition from kesterite to stannite occurs with 60% iron. More recently, the structural transition from kesterite to stannite was, instead, assessed for an amount of iron greater than 75% [14]. The C(Z,F)TS optical band gap is frequently reported as decreasing from 1.5 to 1.2 eV with increasing iron content [13,14,15,16,17]. Solution processing methods have proven to be very suitable to produce CZTS-based solar cells, though they require annealing in a sulfur vapor atmosphere to form the pure polycrystalline phase [15,18,19]. Here, we report a sol-gel procedure with drop-casting deposition to prepare C(Z,F)TS thin films for photovoltaic applications. The sol-gel process employs a DMSO-based solution containing a high concentration of thiourea to not necessitate the addition of sulfur during the annealing step [20]. Herein, we use the molecular ink to produce high-quality thin films, suitable for photovoltaic applications, and by controlling the environment of the ink synthesis and deposition, we manage to obtain high-quality C(Z,F)TS thin films. The 80% and 100% Fe thin films proved to be particularly attractive for use in cheap and easily processable solar cells.

## 2. Materials and Methods

### 2.1. Precursor Solution

The detailed procedure to synthesize the molecular ink was published by us elsewhere [20]. Notably, 0.13 M of a mixture of anhydrous iron acetate Fe(CH_3_COO)_2_ (90% TCI) and zinc acetate dihydrate Zn(CH_3_COO)_2_·2H_2_O (99.99% Sigma-Aldrich, Darmstadt, Germany), 0.12 M tin dichloride dihydrate SnCl_2_·2H_2_O (98% Sigma-Aldrich, Darmstadt, Germany), 0.25 M copper acetate monohydrate Cu(CH_3_COO)_2_·H_2_O (99% Merck, Darmstadt, Germany), and 1.25 M thiourea NH_2_CSNH_2_ (99% Sigma-Aldrich, Darmstadt, Germany) were solved in dimethyl sulfoxide (99.90% Sigma-Aldrich, Darmstadt, Germany) for the samples realized in air and dimethyl sulfoxide (anhydrous, ≥99.9% Sigma-Aldrich, Darmstadt, Germany) for the samples realized in a glovebox.

### 2.2. Thin-Films Synthesis

The deposition method made use of a sol-gel procedure with drop-casting deposition. The deposition took place on fluorine tin oxide (FTO)-coated glass. For the samples produced in air, C(Z,F)TS AIR, the substrate underwent ozone UV-light treatment to increase the adhesion of a 1 μL cm^−2^ drop. After deposition, the samples were left for 5 min in air and were then placed under 10^−1^ mbar vacuum for 20 min to dry. For the samples prepared under a controlled inert atmosphere, C(Z,F)TS GB, a nitrogen-filled glovebox was used, where the precursor solution had been entirely prepared, as described in the previous paragraph. The drop-casting (onto ozone-UV light-cleaned FTO-glass) was performed in a glovebox, and the samples were subsequently put in air to undergo the gelation process (for 30 min). The C(Z,F)TS AIR and C(Z,F)TS GB-coated samples were finally annealed at 500 °C for 1 h. The procedure was repeated once in order to reach a micrometer final film thickness.

### 2.3. Characterization

X-ray diffraction patterns were collected by using a MiniFlex 600, Rigaku (Rigaku, Eschweiler, Germany), equipped with a Cu Kα source (λ = 1.5412 Å). Raman spectroscopy was performed using a Jasco Ventuno micro-Raman spectrometer (Jasco, Cremella, Italy), equipped with a 633 nm laser. XPS was performed on a Kratos Axis Ultra DLD X-ray photoelectron spectrometer (Kratos Analytical Ltd, in Mantchester, U.K.) equipped with a monochromatic Al Kα X-ray source (1486.6 eV), and on a Thermo Scientific Nexsa X-ray photoelectron spectrometer, also with the monochromatic Al Kα X-ray source. The chemical composition of the thin films was studied by the field emission scanning electron microscopy, FE-SEM, Tescan VEGA TS Univac 5136XM (Tescan, Brno, Czech Republic), equipped with an EDS EDAX Genesis 4000 XMS Imaging 60 SEM. The optical band gaps of the C(Z,F)TS thin films were evaluated by measuring transmission and reflectance spectra with a Jasco V-570 UV/Vis/NIR spectrometer (Jasco, Cremella, Italy).

## 3. Results and Discussion

### 3.1. Phase Structure Analysis

**Figure 1:**
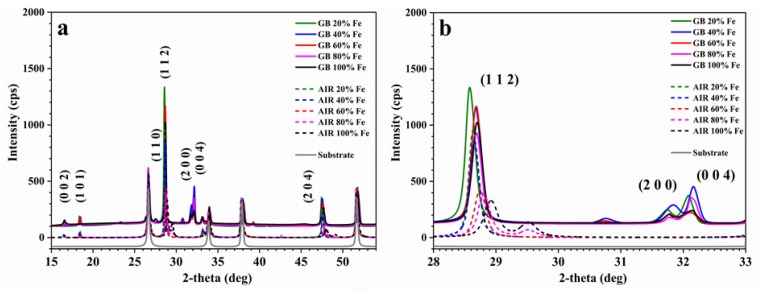
X-ray diffraction (XRD) patterns of the 20%, 40%, 60%, 80%, and 100% CZFTS thin films, made in glovebox and air: (**a**) entire acquisition scale and (**b**) magnification on the main peaks.

The fabricated CZTS thin films were characterized by X-ray diffraction (XRD), and Raman and X-ray photoelectron spectroscopy (XPS). Figure 1 shows XRD patterns of the C(Z,F)TS thin films, varying the amount of iron. The diffraction peaks at 2θ of 16.5°, 18.4°, 27.5°, 28.7°, 31.7°, 32.1°, and 47.6° can be assigned respectively to (002), (101), (110), (112), (004), and (204) crystallographic planes of CZTS and CFTS, in agreement with ICCD No. 01-075-4122 (CZTS) and ICDD No. 00-035-0582 (CFTS) [15].

Both in the samples made in an inert environment and those made in air, there is a peak shift to higher angles, increasing the iron content. In the samples prepared in air, there is also a broadening of the (112) peak when the iron content exceeds 40%, indicating a decrease in the order in the crystallites [15]. Instead, the diffractograms of samples made in the glovebox display narrow peaks, characteristic of highly crystalline thin films. The variation from 28.58° to 28.70° with increasing iron amount is consistent with the literature and is attributed to a decrease in unit cell volume when increasing the iron content [6,8,14,15]. This difference in the two methodologies implies that crystallinity is significantly improved as a result of avoiding exposure to air of the moisture during the preparation of the solutions and the drop-casting procedure.

**Figure 2:**
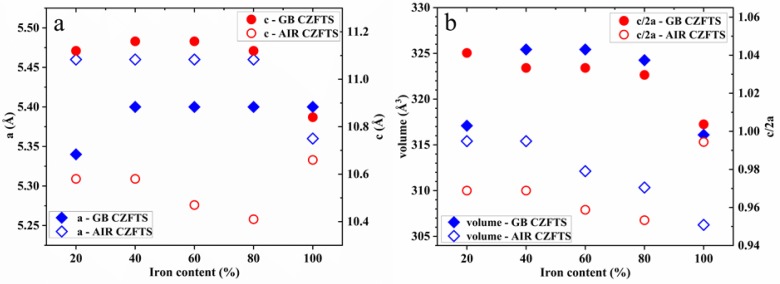
(**a**) Lattice parameters *a* and *c*, calculated by applying the tetragonal structure formula, and (**b**) relative volume *a*^2^*c*, lattice distortion *c/2a* of the 20%, 40%, 60%, 80%, and 100% C(Z,F)TS thin films, made in glovebox and air.

The lattice parameters, *a* and *c*, were calculated by applying the tetragonal structure formula 1/d_hkl_^2^ = (h^2^ + k^2^)/a^2^ + l^2^/c^2^, where (h k l) are the Miller indices and *d* is lattice plane space [21]. In Figure 2, the lattice parameters, the relative unit cell volume *a*^2^*c*, and the tetragonal distortion parameter *c/2a* are summarized. The trends of the lattice parameters *a* and *c* are quite different between the samples made in an inert environment and those prepared in air. Parameter *a* increases for up to 40% iron in GB C(Z,F)TS where it remains almost unchanged upon further increases in the Fe content; meanwhile, in AIR C(Z,F)TS, we register the opposite trend where it remains constant until 80% iron, and then it decreases. Parameter *c* is slightly stretched up to 80% iron, while it falls within the values typically reported in the literature with 100% iron [15,22]. As regards to the samples in air, instead, *c* is stable up to 40% iron, and then the structure is distorted. The variations of parameters *a* and *c*, as a consequence of the cation redistribution in the crystal lattice, are reflected in the volume of the unit cell that decreases with increasing Fe substitution [15,22,23]. The exception is the 20% GB C(Z,F)TS sample, which shows a parameter *a* lower than the others, and its volume is smaller; this is correlated to the partial replacement of Cu with Fe in the reticular sites. The phase structure transition was studied by analyzing the tetragonal distortion parameter: *c/2a* is reported to be higher than 1 for CFTS in the case of the kesterite structure and less than 1 for the stannite structure, and vice versa for CZTS [12,15]. Therefore, we can observe that the samples made in the glovebox have a *c/2a* trend assigned to the distortion in the kesterite phase structure (CFTS *c/2a* = 1.01). The thin films realized in air have a distortion of the crystal lattice that deserves further investigation, but the *c/2a* trend could be attributed to the distortion of the stannite phase (CFTS *c/2a* = 0.99).

**Figure 3:**
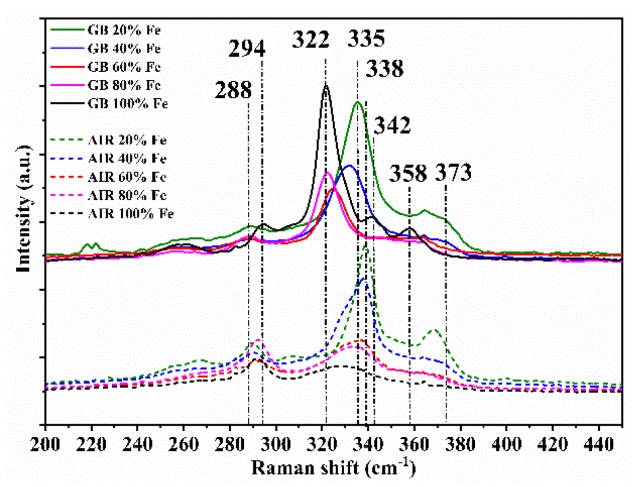
Raman spectra of the 20%, 40%, 60%, 80%, and 100% C(Z,F)TS thin films, made in glovebox and air.

For the samples prepared in a glovebox, *c/2a* decreases abruptly after 20% iron substitution, while for the ones made in air, after 40% iron. At low Fe substitution, Cu is still dominant in the reticular sites of the unit cell base, but with the decrease in the Zn amount, Cu gradually takes the place of Zn, leaving its site to Fe. These atomic rearrangements are characteristic of the structural transition from kesterite to stannite [12,15,24]. In the middle region, *c/2a* slightly changes for the GB C(Z,F)TS films and slowly decreases for AIR C(Z,F)TS films: Here, the double substitution, Cu with Fe and Zn with Cu, leads to the change in crystal parameters. After 80% iron, *c/2a* dramatically decreases in GB C(Z,F)TS films and increases in AIR C(Z,F)TS films, reaching typical values of a stannite structure [15,23]. In XRD patterns, no oxide phase has been detected, but it has been reported that oxygen can compensate a sulfur-deficient chalcogenide stoichiometry, generating the form of Cu_2_(Zn_x_Fe_1−x_)SnO_y_S_4−y_ [15,25,26]. The XRD technique, however, is unable to distinguish some typical secondary phases, because the main peaks of CFTS and CZTS cover those characteristics of some by-products such as ZnS and Cu_2_SnS_3_ [15,27]. Therefore, in order to distinguish the coexistence of secondary phases, the thin films were compared with the support of Raman spectroscopy. Figure 3 shows the Raman spectra of the C(Z,F)TS thin films, varying the iron amount. In the GB 100% Fe film Raman spectrum, the dominant peak is at 322 cm^−1^, and the other peaks are observed at 294 and 358 cm^−1^ [6,15,27]; the peak at 342 cm^−1^ reveals the presence of iron pyrite FeS_2_ [28]; with the decrease in the iron content, the main peak broadens, indicating a shorter coherence length of phonons due to the worsening crystallinity, and moves to higher wavenumbers: The 20% Fe film has the highest peak shift at 335 cm^−1^, which is closer to that of the Kesterite structure, usually observed at 338 cm^−1^ [27,29,30,31]. Moreover, with the decrease in iron content, both the shifting of the peak at 294 to 288 cm^−1^ and the appearance of the peak at 373 cm^−1^, with less than the 40% iron, are consistent with the transition from the stannite to the kesterite phase structure [6,15,27].

The secondary-phase iron pyrite FeS_2_ disappears, adding 20% zinc to CFTS, and producing the stable stannite mineral structure Cu_2_Zn_0.2_Fe_0.8_SnS_4_ [7]. The critical importance of controlling the molecular ink synthesis and deposition is evident in the Raman spectra comparison: Between the samples made in air, only the compounds closest to the kesterite phase, 20% and 40% iron, have good crystallinity, and the appearance of peaks at 290 and 303 cm^−1^, attributed to the ternary phase Cu_2_SnS_3_, is registered [29,31,32].

**Figure 4:**
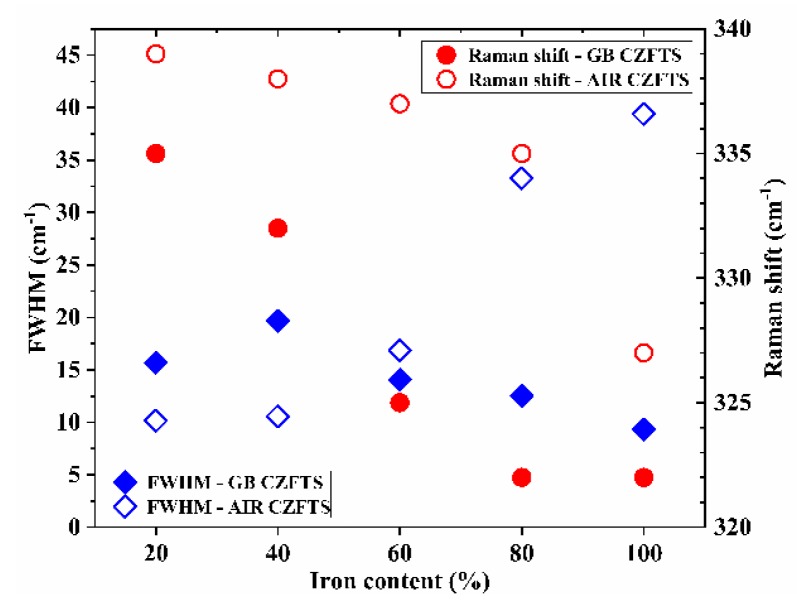
Raman shift position and FWHM of the main peak for the 20%, 40%, 60%, 80%, and 100% C(Z,F)TS thin films, made in glovebox and air.

The full-width at half-maximum, FWHM, of the peaks related to the main vibrational modes depends on the cation order in the lattice [33]. FWHMs were evaluated by the deconvolution of the Raman spectra fitted, with Gaussian or mixed Gaussian–Lorentzian peaks. Figure 4 summarizes the position and the FWHM of the main peak of the C(Z,F)TS thin films. Moving from a peak position still ascribable to kesterite in the GB 20% Fe film, 335 cm^−1^, to an intermediate kesterite phase, such as that of 40%, there is a clear increase in FWHM. The peak width decreases in GB 60% Fe, and it reaches a minimum value of 9.3 cm^−1^ in GB 100% Fe, with a Raman shift typical of the stannite phase [15,27]. The data related to the thin films produced in air confirm the XRD analysis: The main peak in AIR 20% Fe is in the position reported for kesterite structures, and after 40% Fe, the FWHM worsens drastically.

**Figure 5:**
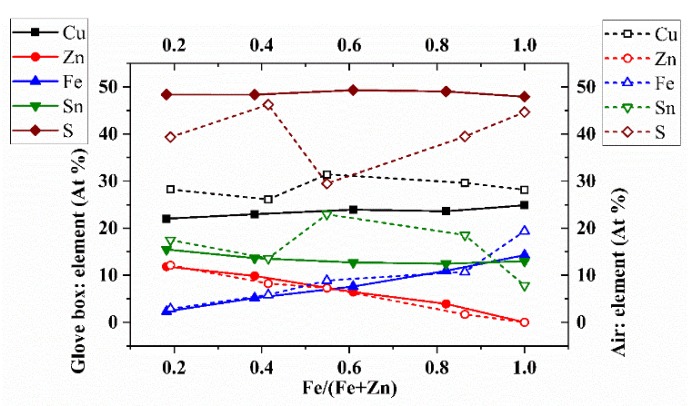
Atomic percentages of Cu, Zn, Fe, Sn, and S, calculated by energy-dispersive spectroscopy, EDS, measurements of the 20%, 40%, 60%, 80%, and 100% C(Z,F)TS thin films, made in glovebox and air, versus the Fe/(Fe+Zn) ratio.

The chemical composition was analyzed through energy-dispersive X-ray spectroscopy, EDS, measurements. The atomic percentages of the components, versus the Fe/(Fe+Zn) ratio, are displayed in Figure 5. The error on the atomic percentage is about 3% on Cu, Zn, Sn, Fe, and S. In the GB thin films, Cu, Sn, and S agree with the values reported in the literature [34]; meanwhile, the AIR thin films show substantial deviations from the expected values. This agrees with the XRD analysis; the distortion in the structure calculated for the AIR samples is reflected in the atomic percentages of Cu, Sn, and S, which are very far from those of the kesterite structure. The EDS analysis reveals that the environment has a substantial impact on the thin-film composition: Only the AIR 40% Fe thin film can be considered in a kesterite composition, also confirming the Raman analysis. The atomic percentages of Fe and Zn follow the same trend in both series, complying with the ratios in the precursor solutions, except for the sample “AIR 100% Fe” that deviates, because, as seen from the Raman analysis, it is mainly composed of the secondary phase Cu_2_SnS_3_, and the iron may be present in abundance in oxidized forms. The EDX confirms a progressive replacement of zinc with iron, with stoichiometric ratios of kesterite up to 74% iron and stannite at 100% iron. The structure rearrangements characteristic of the transition from kesterite to stannite, Cu with Fe and Zn with Cu [12,15,23,24], leads to the crystal parameters distortion hypothesized by XRD analysis for the glovebox samples.

**Figure 6:**
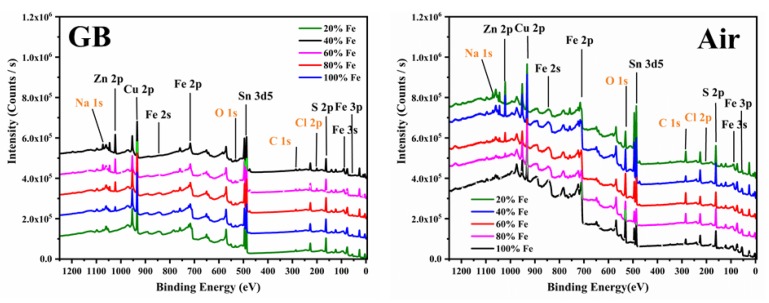
X-ray photoelectron spectroscopy, XPS, survey spectra of the 20%, 40%, 60%, 80%, and 100% C(Z,F)TS thin films, made in glovebox and in air.

The data confirm the XRD and Raman analysis, adding that the operation in a protected environment significantly reduces the formation of secondary phases. The elemental composition of the C(Z,F)TS films was investigated through X-ray photoelectron spectroscopy, XPS, measurements. Figure 6 shows the typical survey spectra of C(Z,F)TS thin films: The peaks corresponding to Cu, Zn, Fe, Sn, and S are identified, and the C 1s and O 1s peaks are also visible. A Na 1s peak appears in all the samples, as inevitable doping from the glass substrate [35]. In the sample processed in air, a decrease in Na content is observed with increasing Fe content: This can be attributed to the increase in thickness that follows the Fe amount in the C(Z,F)TS films grown using the deposition procedure in air. Cl residues of SnCl_2_ are noted in the samples made in gloveboxes in the thin films with percentages of Fe inferior to 40%.

**Figure 7:**
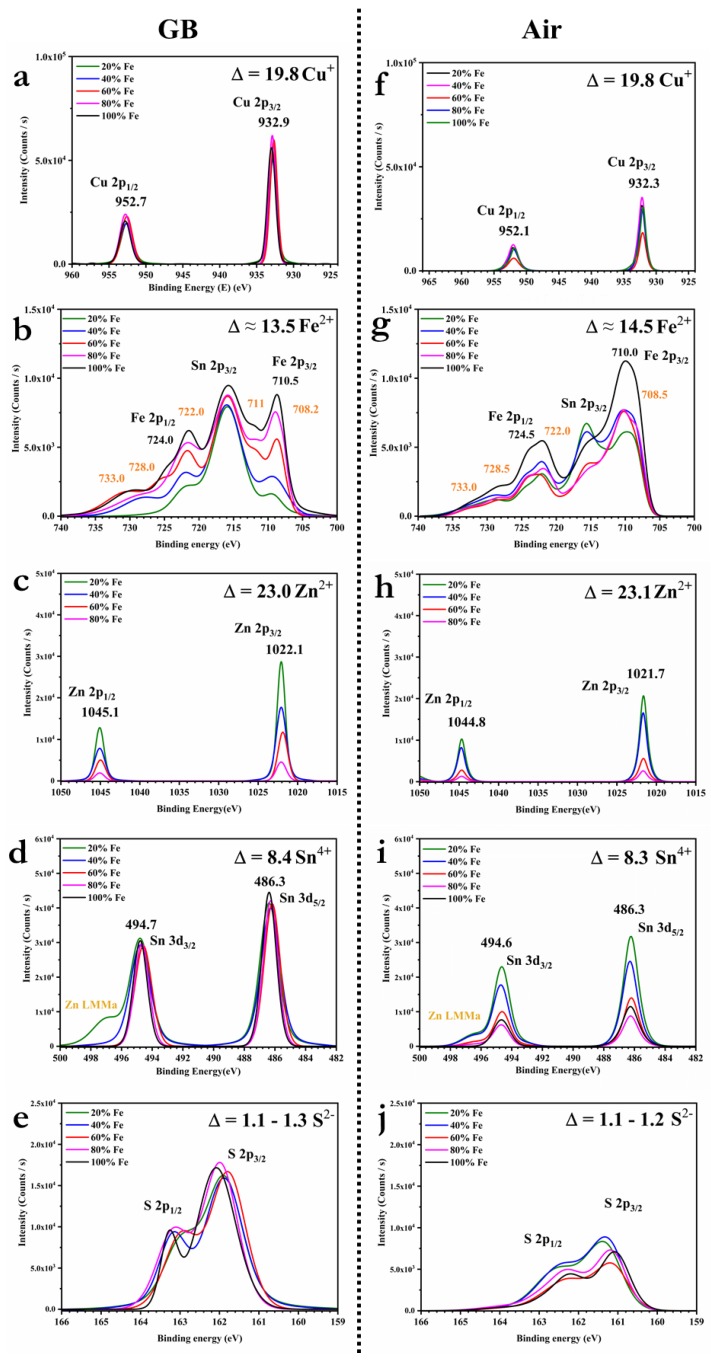
XPS spectra of the high-resolution core level for Cu 2p, Zn 2p, Fe 2p, Sn 3d, and S 2p regions of the 20%, 40%, 60%, 80%, and 100% C(Z,F)TS thin films, made in glovebox (**a**–**e**) and air (**f**–**j**).

**Figure 8:**
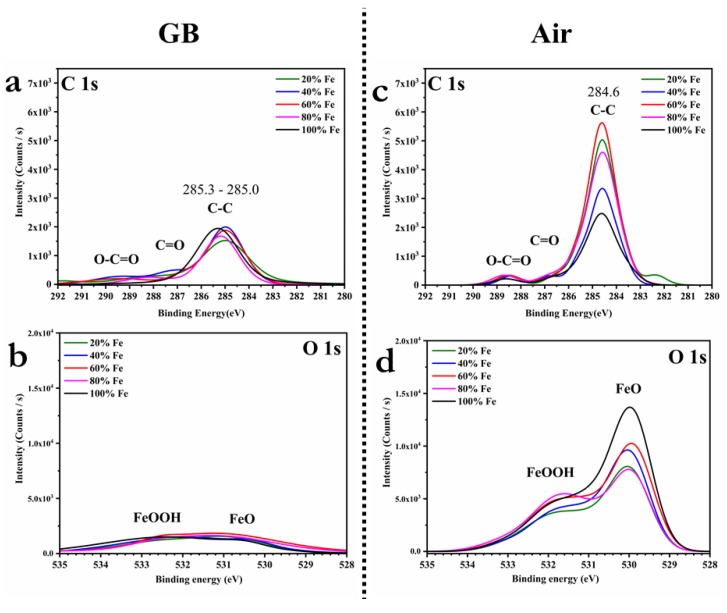
XPS spectra of the high-resolution core level for C 1s and O 1s regions of the 20%, 40%, 60%, 80%, and 100% C(Z,F)TS thin films, made in glovebox (**a**,**b**) and air (**c**,**d**).

High-resolution core-level spectra for Cu 2p, Zn 2p, Fe 2p, Sn 3d, and the S 2p regions were investigated: The spectra are reported in Figure 7, and the fitted parameters of the main peak positions are listed in Table 1. The Cu 2p core-level spectra show that the peaks are located at around 952.3 and 932.5 eV, with a difference of about 19.8 eV between the binding energy of Cu 2p_1/2_ and Cu 2p_3/2_, suggesting the formation of Cu(I) [36,37,38,39]. Typical Fe 2p peaks appeared around 724.2 eV (2p_1/2_) and 710.1 eV (2p_3/2_), confirming the presence of Fe(II). Other peaks are visible at 708, 722, 728, and 733 eV, which can be related to the formation of the chemical species FeS_2_, FeO, FeOOH, and Fe(III) [40]. In C(Z,F)TS prepared in the glovebox, the percentage of Fe (III) is higher, as it can be seen from the onset of the peak at 711 eV in Figure 7b for the sample with more than 60% iron [36]. Recently, it has been suggested that Fe (III) has a clearer doping effect than Fe (II) on CZTS semiconductor layers [14]. The high-resolution core level for the O 1s region, reported in Figure 7c and Figure 8a, confirms the presence of both FeO and FeOOH [41].

The Zn 2p_1/2_ and 2p_3/2_ are visible at binding energies of about 1021.9 and 1044.9 eV, with a peak separation of about 23.0 eV, indicating the presence of Zn(II) [36,37,38,39]. The Sn 3d_3/2_ and Sn 3d_5/2_ peaks are registered around 494.7 and around 486.3 eV, respectively, with a peak separation of about 8.4 eV, confirming the formation of Sn(IV) [36,37,38,39]. The S 2p core-level spectra exhibit the S 2p_1/2_ and S 2p_3/2_ peaks, registered around 162.7 and 161.6 eV, with a peak separation of about 1.1 eV, in agreement with the sulfide phases binding energies [36,37,38,39]. Therefore, in all the analyzed samples, the binding energy for Cu, Zn, Fe, Sn, and S confirmed that the primary chemical states are respectively + 1, + 2, + 2, + 4, and − 2, in agreement with the states in the Cu_2_(Zn,Fe)SnS_4_ chemical formula [19]. Most of the oxygen is bound with the iron, suggesting substitution of S by O in the structure. Therefore, the chemical formula should be better expressed as Cu_2_(Zn_x_Fe_1−x_)SnO_y_S_4−y_ [12,25,26]. The residual carbon is analyzed in Figure 8b,d: The spectra reveal the presence of carbonyls (C=O) and carboxylic acid esters (O–C=O), which most likely originate from adventitious atmospheric carbon dioxide and organic contamination [42,43,44,45]. Comparing the high-resolution core-level spectra of O 1s in Figure 8a,c, the ratio between FeOOH and FeO varies if the samples are made in an inert environment or air, but, above all, the oxide species are overall reduced. The phase structure analysis leads us to conclude that the condensed formula of C(Z,F)TS is more likely Cu_2_(Zn_x_Fe_1−x_)SnO_y_S_4−y_ than Cu_2_(Zn_x_Fe_1−x_)SnS_4_, and in the sample produced in a glovebox, Cu_2_(Zn_0.2_Fe_0.8_)SnO_y_S_4−y_ is in the kesterite phase and Cu_2_FeSnO_y_S_4−y_ is in the stannite structure. We can summarize that kesterite thin films can be produced with up to 20% of iron both in air and in an inert atmosphere, but as soon as the iron content exceeds 40%, the oxidation of the iron dominates and the process has to be carried out in a glovebox. The iron oxidation can be squeezed by controlling the sensitive part of the preparation, i.e., the one in which the components are put in contact in solution to form the precursor ink and the one in which the droplet adheres to the substrate.

### 3.2. Energy Gap Tuning

**Figure 9:**
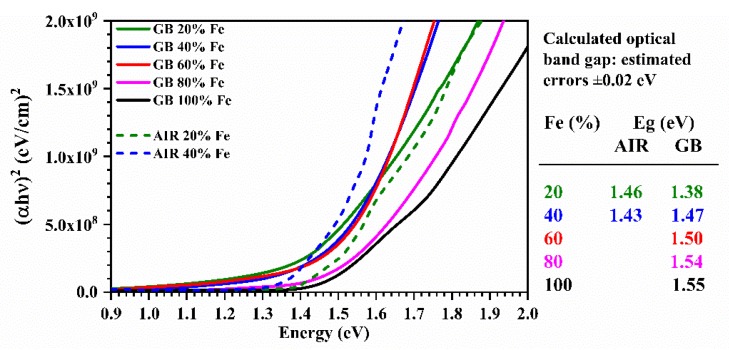
Plot of (α*hν*)^2^ as a function of *hν* for the estimation of the optical bandgap of the 20% and 40% C(Z,F)TS thin films made in air; the 20%, 40%, 60%, 80%, and 100% C(Z,F)TS thin films made in glovebox; and the calculated optical band with estimated errors of ± 0.02 eV.

The optical band range was estimated through UV-Vis spectroscopy measurements; the absorption coefficient (α) was calculated using the relation α(λ) = 1/*t* ln[(1 − R(λ))/T(λ)], where *t* is the thickness of the sample, and T(λ) and R(λ) are the transmittance and reflectance of the film, respectively. The thin films made have α calculated values higher than 1 × 10^4^ cm^−1^. The optical band gap was determined using the relation αℎν = C (ℎν − E_g_), where E_g_ is the bandgap, *h* is the Planck constant, ν is the frequency of the photons, and *C* is a constant. The value of the exponent *n* depends on the nature of the optical transition and, as the C(Z,F)TS is a direct band gap semiconductor [8], the value of n used is 1/2 [46]. Figure 9 shows (α*h*ν)^2^ as a function of *h*ν for samples made in an inert environment and those made in air: The optical band range can be obtained by extrapolating the linear section of the curve and taking the value that intercepts the x-axis [46]. The extrapolated values, with estimated errors of ± 0.02 eV, are reported in Figure 9: The comparison is made between the samples produced in the glovebox and those made in air but with small iron content. The C(Z,F)TS GB dataset is consistent with most of the experimental reports [6,8,14,15,47]. The discrepancy with the C(Z,F)TS AIR thin-film values can be related to a difference in the crystallinity and the stoichiometry [15]. Khadka et al. [15] pointed that in the band gaps reported by Shibuya, they do not take into account the complexity of the conduction band of these compounds, affected by the formation of defects and structural deformations. The 80% and 100% Fe thin films produced in the glovebox prove to be particularly attractive for use in the photovoltaic field, as an efficient photovoltaic semiconductor material has a direct bandgap between 1.1 and 1.8 eV [48,49].

## 4. Conclusions

In this work, the synthesis and characterization of different thin films were made to obtain a material suitable for use in low-cost and environmentally friendly solar cells. The absorber material proposed is based on mixed chalcogenides and composed of economic and non-toxic elements. All thin films were produced by a sol-gel method and deposited by drop-casting, employing a solution containing a large amount of thiourea to not necessitate the addition of sulfur during the annealing step. The phase structure analysis confirmed that by increasing the iron concentration, kesterite evolves to stannite after 80% iron content. By operating the sensitive part of the process in a controlled inert atmosphere, we obtain high-quality samples of C(Z,F)TS thin films. The 80% and 100% Fe thin films proved to be particularly attractive for use in cheap and easily processable solar cells.

## Figures and Tables

**Table 1:** Calculated main peak positions in the XPS spectra of the high-resolution core level for Cu 2p, Zn 2p, Fe 2p, Sn 3d, and S 2p regions of the 20%, 40%, 60%, 80%, and 100% C(Z,F)TS thin films, made in glovebox and air.

Fe (%)	Method	Cu 2p (eV)		Zn 2p (eV)		Fe 2p (eV)		Sn 3d (eV)		S 2p (eV)	
		1/2	3/2	1/2	3/2	1/2	3/2	1/2	3/2	1/2	3/2
20	AIR	951.99	932.29	1044.69	1021.69	723.99	709.79	494.69	486.29	162.49	161.39
	GB	952.68	932.78	1045.08	1022.08	723.88	709.68	494.78	486.38	162.98	161.88
40	AIR	951.65	932.16	1044.76	1021.66	724.16	710.14	494.66	486.26	162.36	161.36
	GB	952.68	932.78	1044.98	1022.08	723.88	709.48	494.78	486.28	163.18	161.88
60	AIR	951.81	932.11	1044.61	1021.71	724.21	710.19	494.61	486.21	162.21	161.21
	GB	952.58	932.68	1044.98	1021.88	725.28	710.98	494.58	486.18	162.88	161.78
80	AIR	951.99	932.09	1044.76	1021.66	724.09	710.29	494.69	486.29	162.29	161.19
	GB	952.78	932.88	1045.08	1022.08	724.08	710.48	494.68	486.28	163.08	161.98
100	AIR	951.85	932.05	/	/	723.85	709.95	494.65	486.25	162.25	161.05
	GB	952.78	933.08	/	/	724.48	710.48	494.78	486.38	163.28	162.08
